# Dyes in modern organic chemistry

**DOI:** 10.3762/bjoc.15.272

**Published:** 2019-11-20

**Authors:** Heiko Ihmels

**Affiliations:** 1 University of Siegen, Department of Chemistry and Biology, and Center of Micro- and Nanochemistry and Engineering, Adolf-Reichwein-Str. 2, 57068 Siegen, Germany

**Keywords:** dyes

Research on organic dyes constitutes a cornerstone in organic chemistry. It started already in 1856 with Perkin's seminal synthesis of mauveine [1]. Since then, dye chemistry evolved into a highly interdisciplinary research area in organic chemistry. Especially the immediate commercialization of Perkin's discovery in a dye firm demonstrated already at that time the strong relationship between research on dyes and industrial chemistry [2–4]. As a result, the success and strength of several chemical companies are originally based on dye chemistry. Although the traditional dyeing of textiles, hair, food, cosmetics, photography or printer colors, etc. is still highly relevant, organic dyes also contribute as essential components in modern applications such as in OLEDs [5–6], solar cells [7–8], organic semiconductors [9], NLO materials [10], photopolymerization [11], or Chemistry 4.0 [12].

From its very beginning, dye chemistry is strongly connected to the scientific developments in organic synthesis. Especially the obvious success and profitability of commercial dyes certainly stimulated the research activities to design and synthesize novel colorants with desirable properties, such as bright and beautiful absorption and emission colors, color fastness, persistence, as well as high molar extinction coefficients and emission quantum yields. Along these lines, numerous new methods and synthetic routes were developed to either functionalize established dye cores or to design new classes of dyes [13–14]. To add to that, efforts were spent to isolate and identify dyes that are found in nature, thus establishing important classes of dyes based on natural products [15–16]. At the same time, dyes themselves turned out to be useful tools in organic synthesis, for example, in traditional photosensitization or in a more recent topical approach as efficient and highly versatile photocatalysts [17–19].

The investigation of dyes is also at the heart of physical organic chemistry. Specifically, the seminal studies to understand the relationship between structure and color are considered highlights in chemistry and contributed significantly to the development of theoretical and computational chemistry [20]. In the same way, essential dye properties, such as aggregation [21–22] or solvatochromism [23], are still in the focus of current physical organic research.

The analytical sciences also depend very much on the availability of appropriate dyes with characteristic properties. Traditionally, organic dyes are the basis of well-established color indicators for qualitative and quantitative analysis. Nowadays, we cannot imagine research in the life sciences or in medical diagnostics without the sophisticated applications of organic dyes as fluorescent probes, dye labels, two-photon absorption probes or chemosensors [24–30].

After all, dye chemistry is literally an "evergreen" in scientific research, and consequently, its significant contributions to scientific developments and breakthroughs is well-documented in several Nobel prizes that are directly or indirectly linked to the synthesis or application of dyes, such as – just to name a few – the ones awarded to A. von Baeyer (1905), H. Fischer (1930), P. Karrer (1937), O. Shimomura, M. Chalfie, R. Tsien (2008), and E. Betzig, S. Hell, W. Moerner (2014).

Although the above mentioned aspects and examples clearly show that dye chemistry is a mature and essential topic in organic chemistry, it still constitutes a vibrant research area because of the continuous need for specifically designed dyes with custom-made optical properties for diverse topical applications. Hence, this special issue aimed at the compilation of the latest trends in the chemistry of organic dyes and it covers the diverse modern aspects with a special focus on organic synthesis, photochemical and photophysical studies, as well as on applications in (bio)analytical chemistry, materials science, biology, and medicine.

I greatly enjoyed acting as guest editor of this thematic issue in the *Beilstein Journal of Organic Chemistry*. With sincere gratitude I would like to thank all authors for their excellent contributions, the referees for their constructive suggestions and the staff of the Beilstein-Institut for their continuous support.

Heiko Ihmels

Siegen, October 2019
